# Are aberrant phase transitions a driver of cellular aging?

**DOI:** 10.1002/bies.201600042

**Published:** 2016-08-24

**Authors:** Simon Alberti, Anthony A. Hyman

**Affiliations:** ^1^ Max Planck Institute of Molecular Cell Biology and GeneticsDresdenGermany

**Keywords:** aging, amyotrophic lateral sclerosis, chaperone, intrinsically disordered protein, mitochondria, neurodegeneration, phase separation, protein aggregation, protein quality control

## Abstract

Why do cells age? Recent advances show that the cytoplasm is organized into many membrane‐less compartments via a process known as phase separation, which ensures spatiotemporal control over diffusion‐limited biochemical reactions. Although phase separation is a powerful mechanism to organize biochemical reactions, it comes with the trade‐off that it is extremely sensitive to changes in physical‐chemical parameters, such as protein concentration, pH, or cellular energy levels. Here, we highlight recent findings showing that age‐related neurodegenerative diseases are linked to aberrant phase transitions in neurons. We discuss how these aberrant phase transitions could be tied to a failure to maintain physiological physical‐chemical conditions. We generalize this idea to suggest that the process of cellular aging involves a progressive loss of the organization of phase‐separated compartments in the cytoplasm.

**Abbreviations**
ALS: amyotrophic lateral sclerosis
FTD: frontotemporal dementia
FUS: fused in sarcoma
HSP70: heat shock protein 70
HSP90: heat shock protein 90
IDP: intrinsically disordered protein
RBP: RNA‐binding protein
TDP‐43: TAR DNA‐binding protein 43

## Introduction

All somatic cells age and die. The process of aging is defined by a progressive decline in physiological function, and is associated with numerous hallmarks, among which are genomic instability, epigenetic alterations, telomere attrition, loss of proteostasis, deregulated signaling, defective intercellular communication, mitochondrial dysfunction, cellular senescence, and stem cell exhaustion 1. These hallmarks of aging can be divided into three distinct categories: (i) the primary hallmarks or causes of cellular aging; (ii) the compensatory or antagonistic responses to cellular aging; and (iii) the integrative hallmarks of aging, which are ultimately responsible for the systemic functional decline 1. Examples of the first category are proteostasis decline and telomere attrition; examples of the second category include cellular senescence and deregulated signaling; examples of the third category include altered intercellular communication and stem cell exhaustion. A major problem is to understand how all these different hallmarks of aging are connected and how they contribute to the overall process of aging.

Despite our limited understanding, it is generally accepted that the cause of aging is a time‐dependent accumulation of cellular damage 2, 3. In young healthy cells, this damage is largely repaired or removed. As cells age, damage increasingly accumulates. This is because in aging cells, homeostatic mechanisms are progressively weakened, and, as a consequence, the cell can no longer repair itself and maintain a functionally healthy state. Thus, at its core, aging is the decline of homeostatic capacity. Many concepts have been proposed to explain this deterioration of homoeostasis with increasing age. One proposal is that widespread damage to biomolecules such as proteins, lipids, or nucleic acids diminishes the homeostatic capability of cells 4. Another idea is that aging involves a gradual decline in metabolic activity, through the loss of mitochondrial function 5, 6, 7, 8. A decline in mitochondrial functions would particularly affect homeostasis mechanisms, because they generally have a high demand for energy. Other concepts include the idea that aging is a consequence of an increase in macromolecular heterogeneity, driven by deregulated gene expression programs 9, 10. However, what remains unclear is how all these diverse molecular defects are associated with aging. Are they driving the process of aging, or are they simple consequences of aging?

Aging ultimately leads to the death of an organism. However, organisms do not generally die of aging, but from the detrimental consequences of one or many age‐related diseases, such as cancer, diabetes, or neurodegenerative disease. This has led to the proposal that aging itself may be a special form of disease or disease complex 11, 12. Others instead see in aging a mostly benign progression of age that simply increases the risk of certain diseases 13. Regardless of whether aging is a disease or not, what is clear is that aging goes along with a universal decline of function that will inevitably lead to the emergence of age‐related diseases. Which of these diseases will eventually cause the death of an organism is highly variable and will depend on the genetic make‐up of the individual and its life history. Although the question as to whether aging is a disease or not will remain controversial, what is clear is that aging and age‐related diseases are intricately linked processes that cannot be separated from each other.

In this review, we explore the possibility that aging‐induced fluctuations in physical‐chemical parameters in cells, driven in part by metabolic decline and a failure of homeostatic systems, may trigger aberrant phase transitions with a consequent loss of control over intracellular organization. We propose that this concept may explain many of the multifaceted hallmarks of aging and the increasing risk of appearance of diseases with age.

## Intracellular organization without membranes

The proper functioning of a cell requires the organization of macromolecules into compartments, which contain and enhance biochemical reactions. Many compartments have no membranes surrounding them. Prominent examples are P granules 14, Cajal bodies 15, 16, nucleoli 17, 18, or paraspeckles 19 in the nucleus, and stress granules 20, 21 and centrosomes 22 in the cytoplasm, and signaling complexes on the cytosolic face of membranes 23, 24.

Emerging evidence over the last decade now shows that many of these compartments are associated with age‐related diseases. As an example, proteins that have been associated genetically with ALS, such as TDP‐43 and FUS, are prominent members of stress granules, paraspeckles, RNA transport granules, and DNA damage repair sites 19, 21, 25, 26, 27. In addition, there is increasing evidence that defects in nuclear compartments such as nucleoli and Cajal bodies are associated with cancer and protein misfolding diseases 28. This suggests that membrane‐less compartments are particularly vulnerable to diseases that are associated with aging.

## Membrane‐less compartments form through phase separation

Why are membrane‐less compartments particularly sensitive to age‐related disease? In the last 5 years cell, biologists have come to understand that formation of these membrane‐less compartments is driven by a process of liquid–liquid demixing 29, 30, 31. The compartments are thought to phase separate from the cytoplasm, leading to the formation of liquid droplets, which stably coexist with their surrounding environment. Prominent examples of compartments that have been shown to be liquids are P granules in *Caenorhabditis elegans* embryos 14, nucleoli 17, and stress granules 21, 32.

Work over the last 5 years has begun to attack the molecular mechanisms behind phase separation. These studies found that intrinsically disordered proteins, or IDPs, are an important class of proteins that drive phase separation 21, 33, 34, 35, 36, 37, 38, 39, 40, 41, 42. More than 30% of the human proteome contains extended regions of intrinsic disorder 43. We do not know yet, but it seems possible that a large fraction of these IDPs are involved in phase separation and the organization of the intracellular environment.

There are different classes of IDPs. One prominent class is devoid of charges and contains polar amino acids (Q, N, S, G) with interspersed aromatic residues (F, Y). These regions of low sequence complexity have been termed prion‐like domains, because of their similarity to proteins in budding yeast that form infectious proteins, or prions 36, 44, 45. The flexibility of the chain, the absence of charges, and recurrent aromatic residues make prion‐like domains very interactive. There are many such proteins in the genome; human cells, for example, contain over 100 prion‐like proteins 36, 45, while in organisms such as the slime mold *Dictyostelium discoideum*, thousands of proteins carry such domains 46, 47. Another class of IDPs is also characterized by low sequence complexity, but these proteins frequently contain amino acids with acidic or basic side chains. One example are RGG repeat containing IDPs such as Ddx4 38. Here, interactions between blocks of charges and aromatic residues play an important role in driving phase separation, which may additionally be regulated by arginine methylation.

Recent findings show that such low complexity IDPs may provide the link between compartment formation and disease. For instance, prion‐like IDPs, such as FUS and TDP‐43, are involved in forming RNA‐containing compartments in the cytoplasm and nucleus, and they are associated with ALS 21, 48, 49, 50, 51, 52. In these cases, the proteins form pathological aggregates in late stages of the disease. Pathological inclusions of FUS and TDP‐43 contain additional RBPs, most notably stress granule proteins, such as the polyA‐binding protein, eIF4G, and TIA‐1 53, 54, 55. These observations suggest that compartments such as stress granules can change their physical properties and slowly mature into pathological aggregates. This raises an important question: what is the relationship between the role of proteins such as FUS and TDP‐43 in compartment formation and their role in disease? Or, to put it another way, why would the liquid‐like nature of membrane‐less compartments make them so vulnerable to disease?

## Liquid phase transition is very sensitive to changes in physical‐chemical conditions

**Figure 1:**
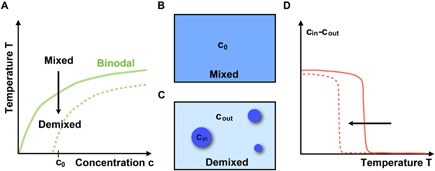
Sensitivity of phase separation to changing environmental conditions. **A:** Phase diagram of a binary mixture as a function of temperature *T* and concentration *c*. **B** and **C:** Upon decreasing the temperature an initially mixed state of mean concentration *c*_0_ demixes, often leading to the formation of liquid droplets. **D:** The concentration of droplets inside minus outside, *c*_in_−*c*_out_, exhibits a jump‐like response when crossing the binodal line in A). Reverting the temperature change or weakly perturbing for example the pH or the salt concentration (dashed line in A and D) can dissolve the drops again.

Liquid–liquid phase separation is a well‐studied phenomenon in soft matter physics. An initially mixed solution of molecules separates into two (or more) phases after, for example, lowering the temperature (Fig. 1A). Typically, droplets of molecules form with an inside concentration that is much larger than the concentration outside of the droplet, in the surrounding milieu (Fig. 1B and C). In general, phase separation is very sensitive to changes in certain parameters, such as temperature, salt, pH, or molecular concentration 29. Droplets form suddenly with a small shift of temperature or salt concentration, and upon reverting the temperature change or weakly perturbing the salt concentration (dashed lines in Fig. 1A and D), droplets can dissolve again. The sensitivity is connected to the steep response of the system, when crossing the binodal line in the phase diagram of Fig. 1A. While the concentration difference between inside and outside of the droplet changes only smoothly upon temperature variations deeply in the mixed and demixed region of the phase diagram, close to the binodal, it shows a steep jump‐like response (Fig. 1D). Therefore, any liquid–liquid phase separation phenomenon will be extremely sensitive to changes in physical‐chemical conditions.

## Aberrant phase transitions are associated with age‐related diseases

Because cellular compartments are formed by phase separation, they must also be sensitive to fluctuating intracellular conditions. Such conditions could, for example, be changes in temperature during heat shock or changes in macromolecular concentration of the phase‐separating molecules. Cellular phase transitions would also be very sensitive to changes in the concentration of ions and small molecules. Indeed, it is now emerging that phase‐separated compartments in living cells are exquisitely sensitive to changes in chemical or physical conditions, such as temperature or simple changes in the concentrations or affinities of macromolecules 18, 23, 24, 29, 38.

**Figure 2:**
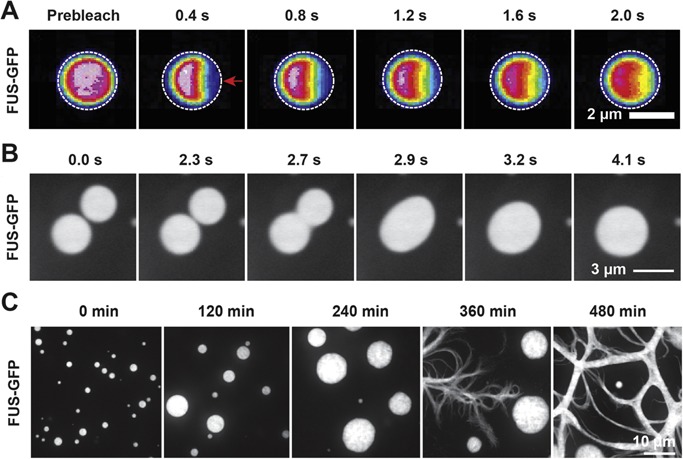
Dynamic behavior of FUS liquid droplets and droplet aging. **A:** Recovery of fluorescence intensity of an in vitro formed FUS–GFP droplet after half‐bleach. The site of photobleach is marked by a red arrow. **B:** Montage of two in vitro reconstituted FUS–GFP droplets fusing under shear flow. **C:** Aging of in vitro reconstituted liquid droplets into fibrous aggregates.

Is this sensitivity to changing conditions also associated with disease? Recent cell‐free experiments with IDPs provide a clue about the relationship between liquid compartments and disease. In most cases liquid compartments quickly convert into more solid‐like structures with different physical properties and structural organization, such as hydrogels and highly ordered fibrils. Proteins that show such behavior are FUS 21, 33, 34, 49 and hnRNPA1 37, 56. Initially, these proteins form liquid‐like assemblies, which fuse, and recover quickly after photobleaching, suggesting rapid dynamics (Fig. 2A and B). However, with time the dynamic properties change, so that they stop fusing and show reduced turnover when examined with photobleaching techniques 21, 37, 49. The material properties of this intermediate state are unclear, but a hydrogel‐like state has been proposed 33, 34. Finally, they can also convert into solid‐like fibrillar aggregates that do not turn over anymore 21, 37 (Fig. 2C). Therefore, there appears to be a driving force for IDPs in liquid‐like compartments to form solids with time. So far, the driving force for solidification is unknown and remains to be clarified. We speculate that it might be accompanied by an outflow of water or could be driven by polymer entanglement that leads to the formation of fibrillar aggregates.

## IDPs can access different phase‐separated material states

So far we have discussed the concept that many membrane‐less compartments are associated with disease. Where they have been looked at, disease‐related compartments form liquid‐like structures in vivo. These diseases are often associated with IDPs, such as prion‐like proteins. When expressed in vitro, these IDPs also form liquid‐like droplets. However, unlike liquid‐like compartments studied in vivo, these liquid‐like droplets rapidly convert into more solid structures such as amyloid‐like fibers.

Several features that enable IDPs to form liquid compartments also make them prone to form solid‐like assemblies. First, IDPs have to be conformationally very flexible to form liquid compartments. Second, they have to be in a state of supersaturation to rapidly respond to changes in conditions. Supersaturation is a state of a solution that contains more dissolved molecules than could be dissolved by the solvent under normal circumstances. When a supersaturated solution demixes into a protein‐poor and a protein‐dense phase, the concentrations of the two phases change until they reach a new equilibrium concentration. Demixing of a supersaturated solution can be triggered through changes in binding affinities of the molecules or changes to the properties of the solvent. Supersaturation and the conformational flexibility of IDPs are necessary requirements for liquid compartment formation, but these two features also make IDPs prone to misfold and form more solid‐like structures.

This, therefore, suggests that a liquid drop is a metastable state, which will quickly convert into a gel or solid‐like state if not regulated. Why would the cell use proteins to form liquid‐like compartments that can so easily transition into pathological solid‐like forms?

One reason why IDPs may be prone to form aberrant solid structures is that cells have also learned to use IDPs to form solid‐like structures with important physiological functions 57. As one can easily imagine, if liquid‐like compartments allow for concentrated biochemical reactions, then solid‐like compartments could be used to inactivate or store macromolecules. Examples of solid‐like structures formed by IDPs are the translational repressor Rim4 in yeast, where an amyloid‐like state appears to regulate gametogenesis 58, and several RNA‐binding proteins that function as molecular memories 59, 60. In oocytes of *Xenopus*, organelles and molecules must be preserved for decades to be then passed on to the next generation. Indeed, *Xenopus* has evolved an amyloid‐like mechanism to form a super‐organelle called the Balbiani body, which stores mRNA and organelles such as mitochondria in quiescent oocytes 61. These physiological amyloids are formed by a prion‐like protein and must have some as yet unknown mechanism that regulates the formation and dissolution of these extremely stable structures.

More generally, dormancy seems to be associated with the transition from a liquid to a solid‐like state. In *C. elegans*, the fluidity of RNP granules in the germ line changes from solid‐ to liquid‐like during the transition from quiescent oocytes to early embryos 62, 63. In budding yeast, there is evidence that numerous proteins assemble into solid‐like structures such as filaments, when yeast cells are challenged with extreme environmental conditions 64, 65, and in bacteria, there is an apparent transition to a glass‐like state upon depletion of ATP 66. Solidification of the cytoplasm has been hypothesized to allow cells to downregulate their metabolism and enter into a dormant state 64, 65.

## A continuum model for phase separating IDPs

**Figure 3:**
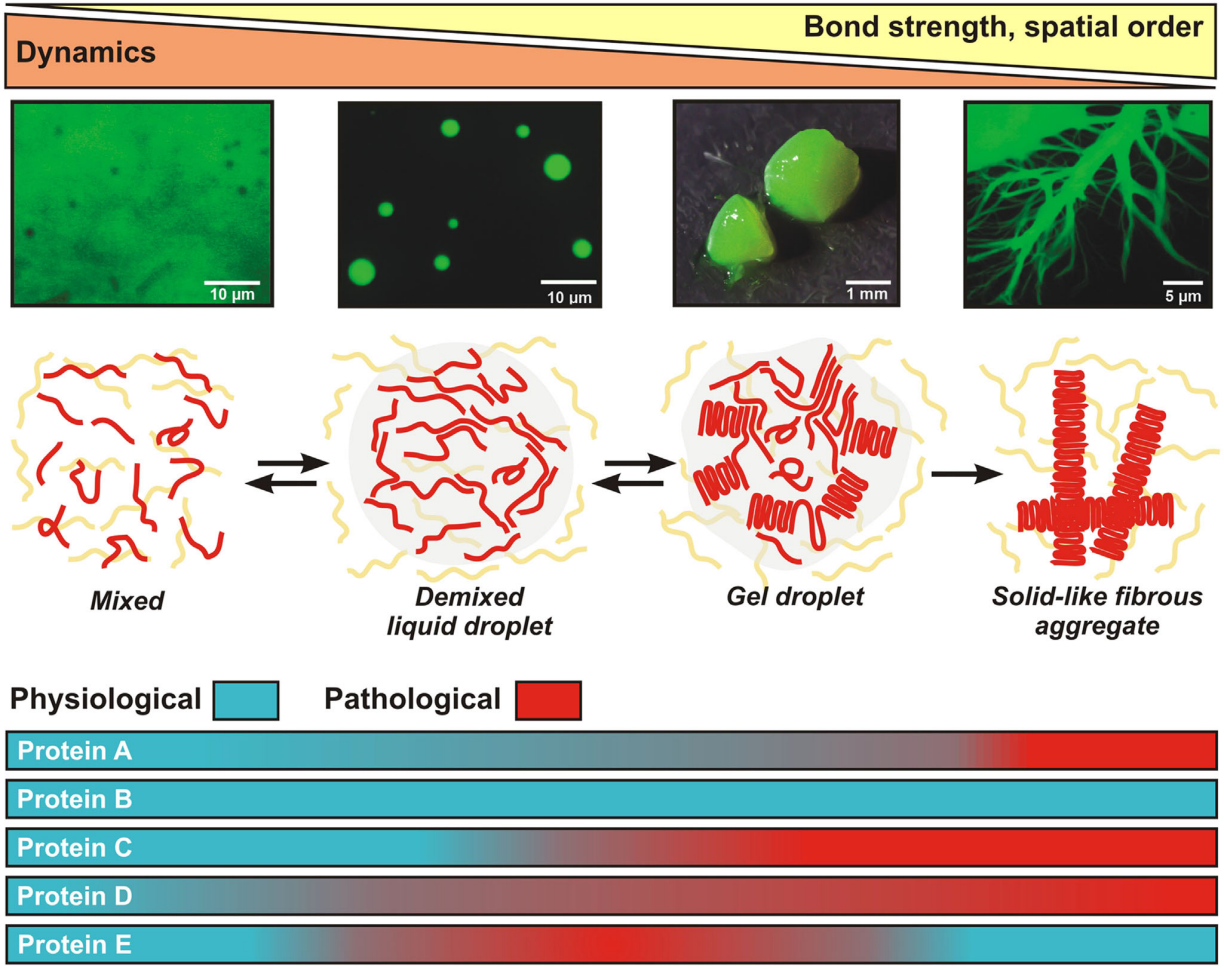
Continuum model of phase separation by intrinsically disordered proteins. An intrinsically disordered protein (shown in red) undergoes a phase transition into a liquid droplet by liquid–liquid demixing. The droplet “ages” with time and adopts different structures and material properties (gel, solid‐like fibrous aggregates). The images on the top show purified FUS protein forming liquid droplets, a gel, and fibrous aggregates 21. Bottom: The hypothetical proteins A–E span the whole range of the continuum. Pathological forms of these proteins are highlighted in red.

We propose that different IDPs adopt different material states and that the material properties of assemblies formed by these IDPs are tuned to purpose (Fig. 3). When biochemical reactions are favored, cells build liquid‐like compartments that concentrate components that can execute diffusion‐limited biochemical reactions. When inactivation or long‐term storage is required, cells shut down biochemical reactions by inducing the formation of solid‐like states that slow diffusion. This must in some way involve tuning of the binding affinities between interacting molecules.

Each phase‐separating protein will have a preferred location on the continuum of material states under physiological conditions, which has been under selection by evolution. However, because many IDPs are exquisitely sensitive to changes in the environment 67, 68, they may access other states along the continuum, when the physicochemical conditions change. Which range of different states a given protein covers will depend on the specific conditions and the evolved molecular properties of the protein. Movements along the continuum may be intended and could be used for sensing stress. This could be achieved by modifying the affinities of interacting IDPs through post‐translational modifications. The fact that IDPs contain many residues such as serine, tyrosine, and arginine, which in principle are modifiable by posttranslational modifications, such as phosphorylation and methylation, suggests that cells make widespread use of such mechanisms.

Therefore, the reason that cells have evolved to use a mechanism that is so prone to error is that it allows tuning of the material properties of their compartments by modulating the binding affinities of IDPs. This comes with the trade off that subtle changes in binding affinities could shift the balance from physiological to pathological assemblies as seen in neurodegeneration. We next discuss mechanisms by which cells could stabilize and maintain metastable liquid‐like states in cells.

## Protein quality control mechanisms regulate phase transitions

Because of this propensity of IDPs to undergo aberrant phase transitions, cells must invest enormous effort to control phase transitions and prevent a conversion into an aberrant state. This is especially true for the liquid‐like compartments, which, as we have discussed above, are likely to be metastable. Indeed, cells have developed numerous quality control or proteostasis systems that regulate and prevent aberrant conformational transitions of IDPs 69. One example are chaperones. These proteins are well known to play a critical role in recognizing and reversing aberrant protein conformational states 70. Chaperones of the HSP70 family alone constitute up to 3% of the protein mass in a cell. These ATP‐driven machines bind aberrantly folded proteins and cooperate with other chaperone systems, such as HSP90, to promote their refolding into a normal state. There also are ATP‐independent chaperones, such as small heat shock proteins, which neutralize aberrant conformations through binding and sequestration. If chaperone systems fail, cells have disposal systems such as the proteasome or autophagy machinery in place, which remove aberrant structures by degradation 71, 72. In fact, there are numerous examples where these systems fail in age‐related diseases. For example, failure of the autophagy machinery is now considered one of the key pathomechanisms in ALS and FTD 71, 73, 74, 75, 76, 77, 78. There are many additional examples of how defects in the proteostasis machinery affect protein conformations and associated diseases, in particular in aging organisms.

## Changes in metabolism have a strong impact on phase transitions

The sensitivity of phase transitions to different metabolic conditions means that a cell must also go to great lengths to control the metabolic state of the cytoplasm and to keep its energy levels constant. Less is known about how defects in this basic parameter is linked to disease. Simple experiments affecting the energy levels of the cell have been shown to alter the viscosity of nucleoli 17 and stress granules 79. Severe changes in energy levels even affect the overall state of the cytoplasm in bacteria, yeast, and *D. discoideum*, by inducing a transition from a fluid to a solid‐like state 65, 66. This indicates that, in living cells, membrane‐less compartments are out of equilibrium systems that require constant energy input to maintain their dynamic properties. The continuous activity of ATP‐driven machines may, for example, be employed to rearrange molecules or loosen up tight interactions that form with time. Indeed, evidence is now emerging that ATP‐driven machines such as helicases and chaperones are intimately involved in regulating the material properties of RNP granules. In the case of the helicase DDX6, inactivation led to the conversion of RNP granules from a disordered liquid‐like state into a well‐ordered crystal‐like assembly 62. In addition, in yeast cells, the recovery from a solid‐like stress granule state depends on an ATP‐driven chaperone, without which cells cannot recover from a solid‐like state of the cytoplasm 32, 80.

We can also envision that the cytoplasm contains many small molecules that tune the phase behavior of proteins and RNA. Trehalose, for example, is produced in great amounts in stressed cells and keeps proteins in a more soluble state 81, 82, 83. Phase‐separating proteins have evolved in the complex environment of the cytoplasm, and therefore depend on the chemical heterogeneity and the specific physical and chemical conditions that characterize the cytoplasm (neutral pH, high potassium and low sodium concentrations inside, high background concentration of macromolecules, small molecules, etc.). Any changes to these parameters will therefore have sweeping consequences for cellular organization and homeostasis. Conversely, cells could use changes in physicochemical conditions such as pH to regulate cellular functions through rapid phase transitions, as has recently been shown for yeast entering into a dormant state 64, 65.

## How aging and age‐related diseases affect RNP granules

In the following, we will take the example of RNA compartments to illustrate how the numerous changes in aging cells could lead to aberrant phase transitions that cause disease and accelerate aging.

### Protein concentration

One trigger of aberrant phase transitions could be changes in macromolecular concentrations or heterogeneity. As discussed before, RNP granules are formed in a concentration‐dependent manner, through phase separation. This process has advantages, but it is also dangerous, because it requires a cell to keep the concentrations of phase‐separating proteins in a defined range. Aging is associated with a loss of control over gene expression and a resulting increase in molecular heterogeneity and changes in macromolecular concentrations 84. This could elevate or reduce the cellular concentration of phase‐separating proteins, with immediate consequences for compartment formation. Increases in heterogeneity could also change the well‐balanced composition of RNP granules, which may be required for maintaining RNP dynamics and functionality. The conformations of IDPs within RNP granules may be controlled through complex formation with other proteins or RNAs, and changes in the concentrations of binding partners may increase the incidence of aberrant conformational states that arise within RNP granules. Aging‐induced changes in molecular heterogeneity could, thus, dramatically shift the phase behavior and physical properties of intracellular compartments. Indeed, when investigated in vitro, the RNA‐binding protein FUS takes up different material properties depending on the concentration of the protein 21. At physiological concentration, it forms droplets that remain liquid for many hours. However, when the protein concentration is raised further or one just waits for a longer time, a gel‐like state develops.

### Nuclear and cytoplasmic localization of proteins

There now is evidence that normal aging as well as neurodegenerative diseases affect the shuttling of many RBPs between the nucleus and the cytoplasm 85, 86, 87, 88, 89. However, maintaining a correct nucleo‐cytoplasmic distribution of RBPs is important, because the nucleus is a very different environment than the cytoplasm. Altered intracellular distributions of RBPs could significantly affect the compositions and properties of RNP granules. In fact, mutations in disease proteins often increase the cytoplasmic and decrease the nuclear concentration of the affected protein 86. A prominent example is FUS, in which most of the ALS‐associated mutations map to the nuclear localization sequence. Direct consequences of these mutations are loss‐of‐function defects in the nucleus, which cause additional problems with transcription, RNA processing, or DNA damage repair 48, 50, 90. However, gain‐of‐function defects have also been observed 49. These gain‐of‐function defects could be related to aberrant phase transitions, which alter the material properties of FUS compartments. Thus, one can imagine that changing the composition and the dynamics of RNP granules through an increased cytoplasmic FUS concentration could cause gain‐of‐function defects through aberrant phase transitions.

### Binding affinities and mutations

Aberrant phase separation could also be induced by changing the binding affinities of interacting IDPs. For example, mutations that increase the propensity of phase‐separating proteins to spontaneously convert into a solid state have been identified in ALS patients. A prominent example is the prion‐like protein TDP‐43, where most of the mutations map to an intrinsically disordered C‐terminal domain that drives the phase behavior of this protein 26. Further examples are FUS and hnRNPA1, which also form solid‐like aggregated states more rapidly when mutated in the prion‐like domain 21, 37, 91. It is possible that many mutations exist in a given proteome that slightly change the phase behavior of affected proteins, but under normal conditions are phenotypically silent. However, when these mutations are placed in the right genetic background, they may unfold devastating effects. This was illustrated by the finding that expansions of a low complexity Q‐rich sequence in ataxin‐2 are usually without effect, but when combined with mutations in the protein TDP‐43 dramatically accelerate the development of a disease 92. We do not know how many such subtle changes exist in a given proteome. In particular, in the late stages of aging, when cells start to lose control over homeostasis, these polymorphisms may increasingly impact compartment formation, with severe effects on cellular function. Changes in the general physical or chemical conditions of the cytoplasm would also strongly affect the phase behavior of RNP granule‐forming proteins. In vitro experiments with RBPs have shown a strong dependence of phase separation on pH, ions and temperatures 21, 33, 34, 37, 38, 91, 93. Thus, any failure of homeostatic mechanisms normally balancing these parameters will have catastrophic effects for RNP granule dynamics and function. Especially in the late stages of aging, cells may struggle to maintain homeostasis, and basic parameters such as ion concentrations and pH would increasingly get out of control.

### Posttranslational modifications and binding partners

Another source of aberrant phase transitions could be a failure of post‐translational mechanisms that normally control the assembly and fluidity of compartments, in particular methylation and phosphorylation 20, 33, 34, 35, 38, 94. Moreover, RBP proteins such as FUS usually function in a complex with polyvalent molecules, such as RNA or poly(ADP) ribose (PAR) 21, 95, 96, 97, 98. Thus, global or local changes in RNA or PAR concentrations could lead to downstream effects, which negatively impact compartment dynamics and function. That such changes have a strong effect on compartments is illustrated by the finding that the nucleolus hardens when transcription is inhibited 99. Once a liquid compartment has turned into a more solid state, additional problems may occur. An increase in the amount of cross‐β sheets may lead to the trapping of proteins, as shown for aberrant assemblies of FUS 49. IDPs often show promiscuous binding behavior and some IDPs exist in very low numbers, suggesting that aberrant trapping of IDPs could be a significant problem, especially for those IDPs that exist in low concentrations and have essential functions. This has been demonstrated recently for TDP‐43 aggregates in the cytoplasm, which trap nucleo‐cytoplasmic shuttling factors, thus causing a breakdown of nuclear transport and potential secondary defects in splicing and transcription 100.

### Misfolded proteins and stress

Chronic stress is another aspect that could accelerate aging through aberrant phase transitions. There is evidence that in stressed cells, RNP granules specifically recruit misfolded proteins such as defective ribosomal products (DRiPs), especially when the protein quality control machinery is overwhelmed 101. IDPs in RNP granules seem to have a tendency to bind misfolding‐prone proteins 32. Trapping of misfolded proteins in RNP granules could promote intra‐compartment cross‐seeding events and subsequent spreading of cross‐β sheet conformations to compartment‐forming proteins, followed by compartment hardening. Failure of protein quality control mechanisms such as ATP‐driven chaperones or disposal mechanisms could further accelerate this hardening reaction and cause additional functional defects.

### Metabolic state and mitochondria

Decline of mitochondrial activity has been commonly linked to aging and age‐related diseases, but it has been hard to pinpoint why. We suggest that, as mitochondrial function declines, changes in the properties of the cytoplasm that are governed by mitochondria, such as regulation of ATP levels or lipid metabolism, will make it harder and harder to properly regulate the numerous homeostasis mechanisms that regulate phase transitions as described above. This will further accelerate the defects commonly seen as problems of aging. Furthermore, there is now substantial evidence of a link between mitochondrial failure and age‐related neurodegeneration 5, 102, 103. Regardless of whether or not mitochondrial defects are a cause or a consequence of these diseases, defective mitochondria will likely accelerate the pathology as phase transitions start to fail.

We can now see why patients with mutations in proteins such as FUS and TDP43 only develop disease later in life. When cells are young, they have an active metabolism that allows them to control the tendency of these proteins to aggregate. However, one can imagine that when mitochondrial activity begins to decline, for instance through the accumulation of somatic mutations, the metabolic potential of the cells will also decrease, thus triggering a loss of homeostasis and aberrant phase transitions. Indeed, it is known that experimentally increasing the rate of mitochondrial mutations in mice will also increase the rate of aging 6. The stochastic nature of age‐related diseases could therefore be due in large parts to the stochastic nature of mitochondrial decline.

As discussed in this review, there are multiple different systems that prevent aberrant phase transitions in young cells. However, they will all decline with increasing age, causing an increasing loss of control over cellular organization. Thus, aberrant phase transitions could be an important driver that accelerates the aging process and causes the onset of age‐related diseases.

## Conclusions

Current research into aging and age‐related diseases is biased toward “omics” approaches. This has generated tremendous insight, but often the underlying molecular causes of aging and age‐related diseases have remained unclear. At its heart, aging is a process of cellular functional decline. In this review, we examined the idea that one aspect of this problem could be the difficulty of cells to properly regulate phase separation as the metabolic activity and the homeostatic capacity of the cells decreases. Because phase separation is involved in numerous different processes in a cell, this would help to explain why cellular aging is such a multifactorial process. Understanding aging and its associated diseases will require us to look at a cell as an out of equilibrium system that maintains its self‐organized, dynamic state through continuous energy consumption, and through incredibly tight control over the concentrations and consistency of its intracellular components.

We have mostly concentrated on neurodegeneration, and in particular on ALS and related aging‐associated diseases, because most experimental evidence on aberrant phase transitions has been generated for these diseases. However, other neurodegenerative disorders have also been tied to IDPs, such Alzheimer's and Parkinson's disease. Moreover, many forms of cancer are triggered by somatic mutations or aggregation of IDPs, one prominent example being the “guardian of the genome” p53 104. It is not hard to imagine that a failure of phase separation and compartment formation may also play important roles in the progression of these and other diseases such as diabetes.

The authors have declared no conflicts of interest.

## References

[1] López‐Otín C , Blasco MA , Partridge L , Serrano M , et al. 2013 The hallmarks of aging. Cell 153: 1194–217. 23746838 10.1016/j.cell.2013.05.039 PMC3836174

[2] Gems D , Partridge L . 2013 Genetics of longevity in model organisms: debates and paradigm shifts. Annu Rev Physiol 75: 621–44. 23190075 10.1146/annurev-physiol-030212-183712

[3] Kirkwood TBL . 2005 Understanding the odd science of aging. Cell 120: 437–47. 15734677 10.1016/j.cell.2005.01.027

[4] Rattan SIS . 2008 Increased molecular damage and heterogeneity as the basis of aging. Biol Chem 389: 267–72. 18208348 10.1515/BC.2008.030

[5] Burté F , Carelli V , Chinnery PF , Yu‐Wai‐Man P . 2015 Disturbed mitochondrial dynamics and neurodegenerative disorders. Nat Rev Neurol 11: 11–24. 25486875 10.1038/nrneurol.2014.228

[6] Bratic A , Larsson N‐G . 2013 The role of mitochondria in aging. J Clin Invest 123: 951–7. 23454757 10.1172/JCI64125 PMC3582127

[7] Jensen MB , Jasper H . 2014 Mitochondrial proteostasis in the control of aging and longevity. Cell Metab 20: 214–25. 24930971 10.1016/j.cmet.2014.05.006 PMC4274350

[8] Sun N , Youle RJ , Finkel T . 2016 The mitochondrial basis of aging. Mol Cell 61: 654–66. 26942670 10.1016/j.molcel.2016.01.028 PMC4779179

[9] Gems D . 2015 The aging‐disease false dichotomy: understanding senescence as pathology. Front Genet 6: 212. 26136770 10.3389/fgene.2015.00212 PMC4468941

[10] Bahar R , Hartmann CH , Rodriguez KA , Denny AD , et al. 2006 Increased cell‐to‐cell variation in gene expression in ageing mouse heart. Nature 441: 1011–4. 16791200 10.1038/nature04844

[11] Gems D . 2011 Tragedy and delight: the ethics of decelerated ageing. Philos. Trans R Soc Lond B Biol Sci 366: 108–12. 21115537 10.1098/rstb.2010.0288 PMC3001315

[12] Bulterijs S , Hull RS , Björk VCE , Roy AG . 2015 It is time to classify biological aging as a disease. Front Genet 6: 205. 26150825 10.3389/fgene.2015.00205 PMC4471741

[13] Rattan SIS . 2014 Aging is not a disease: implications for intervention. Aging Dis 5: 196–202. 24900942 10.14336/AD.2014.0500196 PMC4037311

[14] Brangwynne CP , Eckmann CR , Courson DS , Rybarska A , et al. 2009 Germline P granules are liquid droplets that localize by controlled dissolution/condensation. Science 324: 1729–32. 19460965 10.1126/science.1172046

[15] Strzelecka M , Trowitzsch S , Weber G , Lührmann R , et al. 2010 Coilin‐dependent snRNP assembly is essential for zebrafish embryogenesis. Nat Struct Mol Biol 17: 403–9. 20357773 10.1038/nsmb.1783

[16] Handwerger KE , Cordero JA , Gall JG . 2005 Cajal bodies, nucleoli, and speckles in the Xenopus oocyte nucleus have a low‐density, sponge‐like structure. Mol Biol Cell 16: 202–11. 15509651 10.1091/mbc.E04-08-0742 PMC539164

[17] Brangwynne CP , Mitchison TJ , Hyman AA . 2011 Active liquid‐like behavior of nucleoli determines their size and shape in Xenopus laevis oocytes. Proc Natl Acad Sci USA 108: 4334–9. 21368180 10.1073/pnas.1017150108 PMC3060270

[18] Weber SC , Brangwynne CP . 2015 Inverse size scaling of the nucleolus by a concentration‐dependent phase transition. Curr Biol 25: 641–6. 25702583 10.1016/j.cub.2015.01.012 PMC4348177

[19] Hennig S , Kong G , Mannen T , Sadowska A , et al. 2015 Prion‐like domains in RNA binding proteins are essential for building subnuclear paraspeckles. J Cell Biol 210: 529–39. 26283796 10.1083/jcb.201504117 PMC4539981

[20] Wippich F , Bodenmiller B , Trajkovska MG , Wanka S , et al. 2013 Dual specificity kinase DYRK3 couples stress granule condensation/dissolution to mTORC1 signaling. Cell 152: 791–805. 23415227 10.1016/j.cell.2013.01.033

[21] Patel A , Lee HO , Jawerth L , Maharana S , et al. 2015 A liquid‐to‐solid phase transition of the ALS protein FUS accelerated by disease mutation. Cell 162: 1066–77. 26317470 10.1016/j.cell.2015.07.047

[22] Woodruff JB , Wueseke O , Viscardi V , Mahamid J , et al. 2015 Centrosomes. Regulated assembly of a supramolecular centrosome scaffold in vitro. Science 348: 808–12. 25977552 10.1126/science.aaa3923 PMC5039038

[23] Li P , Banjade S , Cheng H‐C , Kim S , et al. 2012 Phase transitions in the assembly of multivalent signalling proteins. Nature 483: 336–40. 22398450 10.1038/nature10879 PMC3343696

[24] Banjade S , Rosen MK . 2014 Phase transitions of multivalent proteins can promote clustering of membrane receptors. eLife 3: e04123. 10.7554/eLife.04123 PMC4238058 25321392

[25] Alami NH , Smith RB , Carrasco MA , Williams LA , et al. 2014 Axonal transport of TDP‐43 mRNA granules is impaired by ALS‐causing mutations. Neuron 81: 536–43. 24507191 10.1016/j.neuron.2013.12.018 PMC3939050

[26] Li YR , King OD , Shorter J , Gitler AD . 2013 Stress granules as crucibles of ALS pathogenesis. J Cell Biol 201: 361–72. 23629963 10.1083/jcb.201302044 PMC3639398

[27] Mastrocola AS , Kim SH , Trinh AT , Rodenkirch LA , et al. 2013 The RNA‐binding protein fused in sarcoma (FUS) functions downstream of poly(ADP‐ribose) polymerase (PARP) in response to DNA damage. J Biol Chem 288: 24731–41. 23833192 10.1074/jbc.M113.497974 PMC3750169

[28] Sleeman JE , Trinkle‐Mulcahy L . 2014 Nuclear bodies: new insights into assembly/dynamics and disease relevance. Curr Opin Cell Biol 28: 76–83. 24704702 10.1016/j.ceb.2014.03.004

[29] Hyman AA , Weber CA , Jülicher F . 2014 Liquid‐liquid phase separation in biology. Annu Rev Cell Dev Biol 30: 39–58. 25288112 10.1146/annurev-cellbio-100913-013325

[30] Brangwynne CP , Tompa P , Pappu RV . 2015 Polymer physics of intracellular phase transitions. Nat Phys 11: 899–904.

[31] Schmidt HB , Görlich D . 2016 Transport selectivity of nuclear pores, phase separation, and membraneless organelles. Trends Biochem Sci 41: 46–61. 26705895 10.1016/j.tibs.2015.11.001

[32] Kroschwald S , Maharana S , Mateju D , Malinovska L , et al. 2015 Promiscuous interactions and protein disaggregases determine the material state of stress‐inducible RNP granules. eLife 4: e06807. 26238190 10.7554/eLife.06807 PMC4522596

[33] Han TW , Kato M , Xie S , LC Wu , et al. 2012 Cell‐free formation of RNA granules: bound RNAs identify features and components of cellular assemblies. Cell 149: 768–79. 22579282 10.1016/j.cell.2012.04.016

[34] Kato M , Han TW , Xie S , Shi K , et al. 2012 Cell‐free formation of RNA granules: low complexity sequence domains form dynamic fibers within hydrogels. Cell 149: 753–67. 22579281 10.1016/j.cell.2012.04.017 PMC6347373

[35] Kwon I , Kato M , Xiang S , Wu L , et al. 2013 Phosphorylation‐regulated binding of RNA polymerase II to fibrous polymers of low‐complexity domains. Cell 155: 1049–60. 24267890 10.1016/j.cell.2013.10.033 PMC4010232

[36] Malinovska L , Kroschwald S , Alberti S . 2013 Protein disorder, prion propensities, and self‐organizing macromolecular collectives. Biochim Biophys Acta 1834: 918–31. 23328411 10.1016/j.bbapap.2013.01.003

[37] Molliex A , Temirov J , Lee J , Coughlin M , et al. 2015 Phase separation by low complexity domains promotes stress granule assembly and drives pathological fibrillization. Cell 163: 123–33. 26406374 10.1016/j.cell.2015.09.015 PMC5149108

[38] Nott TJ , Petsalaki E , Farber P , Jervis D , et al. 2015 Phase transition of a disordered nuage protein generates environmentally responsive membraneless organelles. Mol Cell 57: 936–47. 25747659 10.1016/j.molcel.2015.01.013 PMC4352761

[39] Toretsky JA , Wright PE . 2014 Assemblages: functional units formed by cellular phase separation. J Cell Biol 206: 579–88. 25179628 10.1083/jcb.201404124 PMC4151146

[40] Quiroz FG , Chilkoti A . 2015 Sequence heuristics to encode phase behaviour in intrinsically disordered protein polymers. Nat Mater 14: 1164–71. 26390327 10.1038/nmat4418 PMC4618764

[41] Decker CJ , Teixeira D , Parker R . 2007 Edc3p and a glutamine/asparagine‐rich domain of Lsm4p function in processing body assembly in *Saccharomyces cerevisiae* . J Cell Biol 179: 437–49. 17984320 10.1083/jcb.200704147 PMC2064791

[42] Gilks N , Kedersha N , Ayodele M , Shen L , et al. 2004 Stress granule assembly is mediated by prion‐like aggregation of TIA‐1. Mol Biol Cell 15: 5383–98. 15371533 10.1091/mbc.E04-08-0715 PMC532018

[43] Oldfield CJ , Dunker AK . 2014 Intrinsically disordered proteins and intrinsically disordered protein regions. Annu Rev Biochem 83: 553–84. 24606139 10.1146/annurev-biochem-072711-164947

[44] Alberti S , Halfmann R , King O , Kapila A , et al. 2009 A systematic survey identifies prions and illuminates sequence features of prionogenic proteins. Cell 137: 146–58. 19345193 10.1016/j.cell.2009.02.044 PMC2683788

[45] King OD , Gitler AD , Shorter J . 2012 The tip of the iceberg: RNA‐binding proteins with prion‐like domains in neurodegenerative disease. Brain Res 1462: 61–80. 22445064 10.1016/j.brainres.2012.01.016 PMC3372647

[46] Malinovska L , Alberti S . 2015 Protein misfolding in Dictyostelium: using a freak of nature to gain insight into a universal problem. Prion 9: 339–46. 26529309 10.1080/19336896.2015.1099799 PMC4964863

[47] Malinovska L , Palm S , Gibson K , Verbavatz J‐M , et al. 2015 Dictyostelium discoideum has a highly Q/N‐rich proteome and shows an unusual resilience to protein aggregation. Proc Natl Acad Sci USA 112: E2620–9. 25941378 10.1073/pnas.1504459112 PMC4443358

[48] Deng H , Gao K , Jankovic J . 2014 The role of FUS gene variants in neurodegenerative diseases. Nat Rev Neurol 10: 337–48. 24840975 10.1038/nrneurol.2014.78

[49] Murakami T , Qamar S , Lin JQ , Schierle GSK , et al. 2015 ALS/FTD mutation‐induced phase transition of FUS liquid droplets and reversible hydrogels into irreversible hydrogels impairs RNP granule function. Neuron 88: 678–90. 26526393 10.1016/j.neuron.2015.10.030 PMC4660210

[50] Vance C , Rogelj B , Hortobágyi T , De Vos KJ , et al. 2009 Mutations in FUS, an RNA processing protein, cause familial amyotrophic lateral sclerosis type 6. Science 323: 1208–11. 19251628 10.1126/science.1165942 PMC4516382

[51] Polymenidou M , Lagier‐Tourenne C , Hutt KR , Bennett CF , et al. 2012 Misregulated RNA processing in amyotrophic lateral sclerosis. Brain Res 1462: 3–15. 22444279 10.1016/j.brainres.2012.02.059 PMC3707312

[52] Wang W‐Y , Pan L , Su SC , Quinn EJ , et al. 2013 Interaction of FUS and HDAC1 regulates DNA damage response and repair in neurons. Nat Neurosci 16: 1383–91. 24036913 10.1038/nn.3514 PMC5564396

[53] Dormann D , Rodde R , Edbauer D , Bentmann E , et al. 2010 ALS‐associated fused in sarcoma (FUS) mutations disrupt transportin‐mediated nuclear import. EMBO J 29: 2841–57. 20606625 10.1038/emboj.2010.143 PMC2924641

[54] Bentmann E , Neumann M , Tahirovic S , Rodde R , et al. 2012 Requirements for stress granule recruitment of fused in sarcoma (FUS) and TAR DNA‐binding protein of 43 kDa (TDP‐43). J Biol Chem 287: 23079–94. 22563080 10.1074/jbc.M111.328757 PMC3391091

[55] Liu‐Yesucevitz L , Bilgutay A , Zhang Y‐J , Vanderweyde T , et al. 2010 Tar DNA binding protein‐43 (TDP‐43) associates with stress granules: analysis of cultured cells and pathological brain tissue. PLoS ONE 5: e13250. 20948999 10.1371/journal.pone.0013250 PMC2952586

[56] Kim HJ , Kim NC , Wang Y‐D , Scarborough EA , et al. 2013 Mutations in prion‐like domains in hnRNPA2B1 and hnRNPA1 cause multisystem proteinopathy and ALS. Nature 495: 467–73. 23455423 10.1038/nature11922 PMC3756911

[57] Newby GA , Lindquist S . 2013 Blessings in disguise: biological benefits of prion‐like mechanisms. Trends Cell Biol 23: 251–9. 23485338 10.1016/j.tcb.2013.01.007

[58] Berchowitz LE , Kabachinski G , Walker MR , Carlile TM , et al. 2015 Regulated formation of an amyloid‐like translational repressor governs gametogenesis. Cell 163: 406–18. 26411291 10.1016/j.cell.2015.08.060 PMC4600466

[59] Caudron F , Barral Y . 2013 A super‐assembly of Whi3 encodes memory of deceptive encounters by single cells during yeast courtship. Cell 155: 1244–57. 24315096 10.1016/j.cell.2013.10.046

[60] Si K , Choi Y‐B , White‐Grindley E , Majumdar A , et al. 2010 Aplysia CPEB can form prion‐like multimers in sensory neurons that contribute to long‐term facilitation. Cell 140: 421–35. 20144764 10.1016/j.cell.2010.01.008

[61] Boke E , Ruer M , Wühr M , Coughlin M , et al. 2016 Amyloid‐like self‐assembly of a cellular compartment. Cell 166:637–50. 27471966 10.1016/j.cell.2016.06.051 PMC5082712

[62] Hubstenberger A , Noble SL , Cameron C , Evans TC . 2013 Translation repressors, an RNA helicase, and developmental cues control RNP phase transitions during early development. Dev Cell 27: 161–73. 24176641 10.1016/j.devcel.2013.09.024 PMC3869996

[63] Hubstenberger A , Cameron C , Noble SL , Keenan S , et al. 2015 Modifiers of solid RNP granules control normal RNP dynamics and mRNA activity in early development. J Cell Biol 211: 703–16. 26527741 10.1083/jcb.201504044 PMC4639854

[64] Petrovska I , Nüske E , Munder MC , Kulasegaran G , et al. 2014 Filament formation by metabolic enzymes is a specific adaptation to an advanced state of cellular starvation. eLife 3: e02409. 10.7554/eLife.02409 PMC4011332 24771766

[65] Munder MC , Midtvedt D , Franzmann T , Nüske E , et al. 2016 A pH‐driven transition of the cytoplasm from a fluid‐ to a solid‐like state promotes entry into dormancy. eLife 5: e09347. 27003292 10.7554/eLife.09347 PMC4850707

[66] Parry BR , Surovtsev IV , Cabeen MT , O'Hern CS , et al. 2014 The bacterial cytoplasm has glass‐like properties and is fluidized by metabolic activity. Cell 156: 183–94. 24361104 10.1016/j.cell.2013.11.028 PMC3956598

[67] Ciryam P , Kundra R , Morimoto RI , Dobson CM , et al. 2015 Supersaturation is a major driving force for protein aggregation in neurodegenerative diseases. Trends Pharmacol Sci 36: 72–7. 25636813 10.1016/j.tips.2014.12.004 PMC4643722

[68] Ciryam P , Tartaglia GG , Morimoto RI , Dobson CM , et al. 2013 Widespread aggregation and neurodegenerative diseases are associated with supersaturated proteins. Cell Rep 5: 781–90. 24183671 10.1016/j.celrep.2013.09.043 PMC3883113

[69] Kim YE , Hipp MS , Bracher A , Hayer‐Hartl M , et al. 2013 Molecular chaperone functions in protein folding and proteostasis. Annu Rev Biochem 82: 323–55. 23746257 10.1146/annurev-biochem-060208-092442

[70] Hartl FU , Bracher A , Hayer‐Hartl M . 2011 Molecular chaperones in protein folding and proteostasis. Nature 475: 324–32. 21776078 10.1038/nature10317

[71] Majcher V , Goode A , James V , Layfield R . 2015 Autophagy receptor defects and ALS‐FTLD. Mol Cell Neurosci 66: 43–52. 25683489 10.1016/j.mcn.2015.01.002

[72] Hipp MS , Park S‐H , Hartl FU . 2014 Proteostasis impairment in protein‐misfolding and ‐aggregation diseases. Trends Cell Biol 24: 506–14. 24946960 10.1016/j.tcb.2014.05.003

[73] Buchan JR , Kolaitis R‐M , Taylor JP , Parker R . 2013 Eukaryotic stress granules are cleared by autophagy and Cdc48/VCP function. Cell 153: 1461–74. 23791177 10.1016/j.cell.2013.05.037 PMC3760148

[74] Maruyama H , Morino H , Ito H , Izumi Y , et al. 2010 Mutations of optineurin in amyotrophic lateral sclerosis. Nature 465: 223–6. 20428114 10.1038/nature08971

[75] Ramaswami M , Taylor JP , Parker R . 2013 Altered ribostasis: RNA‐protein granules in degenerative disorders. Cell 154: 727–36. 23953108 10.1016/j.cell.2013.07.038 PMC3811119

[76] Cirulli ET , Lasseigne BN , Petrovski S , Sapp PC , et al. 2015 Exome sequencing in amyotrophic lateral sclerosis identifies risk genes and pathways. Science 347: 1436–41. 25700176 10.1126/science.aaa3650 PMC4437632

[77] Mizuno Y , Amari M , Takatama M , Aizawa H , et al. 2006 Immunoreactivities of p62, an ubiqutin‐binding protein, in the spinal anterior horn cells of patients with amyotrophic lateral sclerosis. J Neurol Sci 249: 13–8. 16820172 10.1016/j.jns.2006.05.060

[78] Deng H‐X , Chen W , Hong S‐T , Boycott KM , et al. 2011 Mutations in UBQLN2 cause dominant X‐linked juvenile and adult‐onset ALS and ALS/dementia. Nature 477: 211–5. 21857683 10.1038/nature10353 PMC3169705

[79] Jain S , Wheeler JR , Walters RW , Agrawal A , et al. 2016 ATPase‐modulated stress granules contain a diverse proteome and substructure. Cell 164: 487–98. 26777405 10.1016/j.cell.2015.12.038 PMC4733397

[80] Cherkasov V , Hofmann S , Druffel‐Augustin S , Mogk A , et al. 2013 Coordination of translational control and protein homeostasis during severe heat stress. Curr Biol 23: 2452–62. 24291094 10.1016/j.cub.2013.09.058

[81] Tapia H , Koshland DE . 2014 Trehalose is a versatile and long‐lived chaperone for desiccation tolerance. Curr Biol 24: 2758–66. 25456447 10.1016/j.cub.2014.10.005

[82] Erkut C , Penkov S , Khesbak H , Vorkel D , et al. 2011 Trehalose renders the dauer larva of *Caenorhabditis elegans* resistant to extreme desiccation. Curr Biol 21: 1331–6. 21782434 10.1016/j.cub.2011.06.064

[83] Singer MA , Lindquist S . 1998 Multiple effects of trehalose on protein folding in vitro and in vivo. Mol Cell 1: 639–48. 9660948 10.1016/s1097-2765(00)80064-7

[84] Walther DM , Kasturi P , Zheng M , Pinkert S , et al. 2015 Widespread proteome remodeling and aggregation in aging *C. elegans* . Cell 161: 919–32. 25957690 10.1016/j.cell.2015.03.032 PMC4643853

[85] Jovičić A , Mertens J , Boeynaems S , Bogaert E , et al. 2015 Modifiers of C9orf72 dipeptide repeat toxicity connect nucleocytoplasmic transport defects to FTD/ALS. Nat Neurosci 18: 1226–9. 26308983 10.1038/nn.4085 PMC4552077

[86] Edbauer D , Haass C . 2015 An amyloid‐like cascade hypothesis for C9orf72 ALS/FTD. Curr Opin Neurobiol 36: 99–106. 26555807 10.1016/j.conb.2015.10.009

[87] Freibaum BD , Lu Y , Lopez‐Gonzalez R , Kim NC , et al. 2015 GGGGCC repeat expansion in C9orf72 compromises nucleocytoplasmic transport. Nature 525: 129–33. 26308899 10.1038/nature14974 PMC4631399

[88] Zhang K , Donnelly CJ , Haeusler AR , Grima JC , et al. 2015 The C9orf72 repeat expansion disrupts nucleocytoplasmic transport. Nature 525: 56–61. 26308891 10.1038/nature14973 PMC4800742

[89] Mertens J , Paquola ACM , Ku M , Hatch E , et al. 2015 Directly reprogrammed human neurons retain aging‐associated transcriptomic signatures and reveal age‐related nucleocytoplasmic defects. Cell Stem Cell 17: 705–18. 26456686 10.1016/j.stem.2015.09.001 PMC5929130

[90] Kwiatkowski TJ, Jr. , Bosco DA , Leclerc AL , Tamrazian E , et al. 2009 Mutations in the FUS/TLS gene on chromosome 16 cause familial amyotrophic lateral sclerosis. Science 323: 1205–8. 19251627 10.1126/science.1166066

[91] Lin Y , Protter DSW , Rosen MK , Parker R . 2015 Formation and maturation of phase‐separated liquid droplets by RNA‐binding proteins. Mol Cell 60: 208–19. 26412307 10.1016/j.molcel.2015.08.018 PMC4609299

[92] Elden AC , Kim H‐J , Hart MP , Chen‐Plotkin AS , et al. 2010 Ataxin‐2 intermediate‐length polyglutamine expansions are associated with increased risk for ALS. Nature 466: 1069–75. 20740007 10.1038/nature09320 PMC2965417

[93] Burke KA , Janke AM , Rhine CL , Fawzi NL . 2015 Residue‐by‐residue view of in vitro FUS granules that bind the C‐terminal domain of RNA polymerase II. Mol Cell 60: 231–41. 26455390 10.1016/j.molcel.2015.09.006 PMC4609301

[94] Dormann D , Madl T , Valori CF , Bentmann E . 2012 Arginine methylation next to the PY‐NLS modulates transportin binding and nuclear import of FUS. EMBO J 31: 4249–370. 22968170 10.1038/emboj.2012.261 PMC3501225

[95] Zhang H , Elbaum‐Garfinkle S , Langdon EM , Taylor N , et al. 2015 RNA controls polyQ protein phase transitions. Mol Cell 60: 220–30. 26474065 10.1016/j.molcel.2015.09.017 PMC5221516

[96] Elbaum‐Garfinkle S , Kim Y , Szczepaniak K , Chen CC‐H , et al. 2015 The disordered P granule protein LAF‐1 drives phase separation into droplets with tunable viscosity and dynamics. Proc Natl Acad Sci USA 112: 7189–94. 26015579 10.1073/pnas.1504822112 PMC4466716

[97] Teloni F , Altmeyer M . 2015 Readers of poly(ADP‐ribose): designed to be fit for purpose. Nucleic Acids Res 44: 993–1006. 26673700 10.1093/nar/gkv1383 PMC4756826

[98] Altmeyer M , Neelsen KJ , Teloni F , Pozdnyakova I , et al. 2015 Liquid demixing of intrinsically disordered proteins is seeded by poly(ADP‐ribose). Nat Commun 6: 8088. 26286827 10.1038/ncomms9088 PMC4560800

[99] Louvet E , Yoshida A , Kumeta M , Takeyasu K . 2013 Probing the stiffness of isolated nucleoli by atomic force microscopy. Histochem Cell Biol 141: 365–81. 24297448 10.1007/s00418-013-1167-9

[100] Woerner AC , Frottin F , Hornburg D , Feng LR , et al. 2016 Cytoplasmic protein aggregates interfere with nucleocytoplasmic transport of protein and RNA. Science 351: 173–6. 26634439 10.1126/science.aad2033

[101] Seguin SJ , Morelli FF , Vinet J , Amore D , et al. 2014 Inhibition of autophagy, lysosome and VCP function impairs stress granule assembly. Cell Death Differ 21: 1838–51. 25034784 10.1038/cdd.2014.103 PMC4227144

[102] Knott AB , Perkins G , Schwarzenbacher R , Bossy‐Wetzel E . 2008 Mitochondrial fragmentation in neurodegeneration. Nat Rev Neurosci 9: 505–18. 18568013 10.1038/nrn2417 PMC2711514

[103] Lin MT , Beal MF . 2006 Mitochondrial dysfunction and oxidative stress in neurodegenerative diseases. Nature 443: 787–95. 17051205 10.1038/nature05292

[104] Wang G , Fersht AR . 2015 Propagation of aggregated p53: cross‐reaction and coaggregation vs. seeding. Proc Natl Acad Sci USA 112: 2443–8. 25675527 10.1073/pnas.1500262112 PMC4345553

